# Efficacy of a strength-based exercise program in patients with chronic tension type headache: a randomized controlled trial

**DOI:** 10.3389/fneur.2023.1256303

**Published:** 2023-09-18

**Authors:** Daniel Martín-Vera, Alberto Sánchez-Sierra, Ángel González-de-la-Flor, Guillermo García-Pérez-de-Sevilla, Diego Domínguez-Balmaseda, Jose Ángel del-Blanco-Muñiz

**Affiliations:** ^1^Department of Physiotherapy, Faculty of Sports Sciences, Universidad Europea de Madrid, Therapeutic Exercise and Fucntional Rehabiltiation Research Group, Villaviciosa de Odón, Madrid, Spain; ^2^Faculty of Biomedical Sciences and Health, European University of Madrid, Villaviciosa de Odón, Madrid, Spain

**Keywords:** tension-type headache, strength exercise, rehabilitation, pain, muscle

## Abstract

**Background:**

Strength-based exercise is widely used to treat tension-type headache, but the evidence of its benefit is unclear. This study aims to analyze the efficacy of a strength-based exercise program in patients with chronic tension-type headaches.

**Methods:**

A randomized controlled trial with a 12-week strength-based exercise program, with chronic tension-type headache. The headache characteristics (which were the primary outcomes: frequency, duration, and intensity), cervical muscle thickness at rest or contraction of multifidus and longus-colli muscle, cervical range of motion, pain pressure threshold of temporalis, upper trapezius, masseter, tibialis muscle and median nerve, and cervical craniocervical flexion test were assessed at baseline and 12-weeks of follow-up in the intervention group (*n* = 20) and the control group (*n* = 20) was performed on 40 patients (85% women, aged 37.0 ± 13.3 years).

**Results:**

Between baseline and week-12 of follow-up the intervention group showed statistically significant differences compared to control group in the following primary outcomes: duration and intensity of headaches. In addition, the intervention group improved the thickness of deep cervical muscles, reduced the peripheral sensitization, and improved the strength of deep cervical flexors.

**Conclusion:**

A 12-week strength training of neck and shoulder region induced changes in pain intensity and duration, and physical-related factors in patients with TTH. Future interventions are needed to investigate if normalization of pain characteristics and physical factors can lead to an increase of headache-related impact.

## Introduction

Headache is defined as a painful sensation of variable intensity located in the cranial vault, the upper part of the neck or nape, and the upper half of the face. According to the International Headache Society (IHS) classification (third edition), tension-type headache (TTH) is considered the most frequent primary headache (1). The lifetime prevalence rate of TTH was around 26% in the general population (2). TTH affects people in activities of daily living in many areas, leading to increased stress levels, impaired motor skills, such as reduced cervical range of motion (ROM) (3), and a negative impact on sleep quality (4). According to the World Health Organization, TTH is one of the 10 most disabling disorders, for both women and men, with a ratio of 3 to 2, respectively. Worldwide, the prevalence of headaches in adults is estimated to be around 50% (5). TTH can be categorized into three subtypes according to the Classification International Headache Disorders (ICHD) based on headache frequency: 1. Infrequent episodic TTH <12 days/year; 2. Frequent episodic TTH 12–180 days/year; 3. Chronic type TTH ≥180 days/year. Currently, according to the third edition of ICHD the diagnosis of TTH is difficult because it shares some of the symptoms with other types of headaches (1, 6). People with chronic daily headaches have an extremely low quality of life in all domains except purely physical or motor functioning, which is less affected (7). Likewise, TTH is associated with a greater number of days of sick leave and a lower quality of social and family relationships (8). These consequences seem to be directly proportional to the duration, frequency, and intensity of the pain (9). TTH occurs due to inflammation or distortion (traction or compression) of pain-sensitive brain structures. The most foundational driver in the etiology of headaches is the musculoskeletal tissue, although we also find other tissues such as arteries and periosteum of the skull, some parts of the dura mater of the base of the skull and the arteries of the dura mater and the pia mater. At the nervous level we find the trigeminal, glossopharyngeal, vagus, and the first three cranial nerves. The bone structure, the parts of the dura mater of the convexity of the skull, the brain parenchyma, and the choroid plexuses are insensitive to pain (10). From all this it can be deduced that the mechanisms that produce pain in headaches are the following: Spasm of the cervical muscles, traction, or dilation of intracranial or extracranial arteries, traction or displacement of the intracranial veins, compression, traction, or inflammation of the cranial or spinal nerves, and meningeal irritation and increased intracranial pressure (10). Muscle spasm is one of the pain mechanisms that triggers pain (11). Within this pathophysiological approach, myofascial pain attributed to referred pain from trigger points in the head and neck muscles is also described (12). For diagnosis and treatment, the Headache Study Group of the Spanish Neurology Society (GECSEN) established a series of consensus recommendations with the aim of categorizing headache episodes with scales that quantify the impact and intensity of headache and disability (13). Regarding the physical examination, it is evaluated through palpation of the painful muscles reported by the patient, such as the frontal, temporal, masseter, pterygoid, sternocleidomastoid, splenius, trapezius (14, 15). The most widely used treatment, published by The American Medical Association for patients with TTH, is pharmacological: simple analgesics and non-steroidal anti-inflammatory drugs are the most recommended (16).

We found scientific evidence that could support the physical therapy in the management of some forms of headaches (17). Among the different conservative therapies, manual therapy is described, achieving positive effects on the intensity and frequency of pain, disability, impact, quality of life and range of craniocervical movement in adults with TTH. Currently, scientific literature suggests the combination of manual therapy and therapeutic exercise to promote an improvement in the symptoms of TTH. Although to date no common consensus has been found, to find the gold standard treatment, and one that can be rigorously replicated for the scientific community (18, 19). The use of therapeutic exercise has shown a decrease in the frequency, duration, and intensity of headaches up to 3/6 months after treatment. In addition, a relationship between deep neck flexor muscle weakness and chronic TTH has been identified (20, 21). Strength training of the deep neck flexor muscles results in an improvement in the intensity and number of TTH episodes by reducing the extensor activity of the suboccipital muscles through mutual inhibition, to restore the afferent inputs of the mechanoreceptors (21, 22).

This study aims to analyze the efficacy of a therapeutic exercise program based on resistance exercises in patients diagnosed with chronic TTH. It also intends to analyze the range of joint mobility and for the first time the changes at the sensory level related to the mechanical sensitivity of different structures will be analyzed bilaterally due to sensory involvement on both sides presented by patients with neuropathic characteristics (23). We hypothesized that supervised 12-week strength training (superiority trial) improves the headaches’ pain intensity, duration, and frequency, as well as the cervical ROM, cervical muscle thickness, craniocervical muscle endurance, and mechanical sensitivity.

## Methods

### Study design

A randomized controlled trial with two parallel groups, an intervention arm and a control arm were carried out following the CONSORT guidelines. This was a superiority trial, as we wanted to show the superiority of a novel strength training intervention.

### Ethical considerations

The current study was approved by the Research Ethics Committee. The study design was registered on 24/05/2022 in the Clinical Trials Registry with the id: ACTRN12622000746730. This study respected the 1964 Helsinki guidelines throughout the study. In addition, all the participants read and signed the informed consent form before being part of this study.

### Participants

Participants were recruited using advertising in a university from June to September 2022. The inclusion criteria to participate in the study were the following: (1) Adults aged 18–65 years; (2) Having chronic TTH for more than 6 months, diagnosed with chronic TTH by their neurologist, following the criteria of the International Headache Society (IHS) classification of headaches, in its third edition. The exclusion criteria were: (1) Presence of pathologies that prevent the performance of physical activity; (2) Pregnancy.

The investigators evaluated eligibility, obtained informed consent, and enrolled the participants. Enrolment ceased when the target sample size was obtained, assuming a loss of 15% of the sample, as recommended by the main investigators.

In the trial registration, we included patients with TTH and migraine, but finally we only included patients with TTH.

#### Sample size

To calculate the sample size, *a priori* power analysis with a two-tailed hypothesis was conducted, with an alpha error of 0.05 and a beta error of 0.2 and medium effect size (*f* = 0.25 or Eta partial squared = 0.06). Therefore, using the G*Power (3.1.9.2) software, a total of 34 participants were required to achieve our objective.

### Randomization

The randomization of the participants was carried out with a computer program (Research Randomizer) by an assistant independent of the study, with a 1:1 ratio, without restriction (simple randomization). This assistant delivered a sequentially numbered sealed envelope with the group to which each participant belonged (control group (CG) or intervention group (IG)) to each participant.

### Blinding

The participants and the investigator who performed the intervention were not blinded.

The outcome assessors were blinded, as well as the statisticians who performed the data analysis.

### Variables

Anthropometric variables are height in centimeters (cm), and weight in kilograms (kg). Height was measured with a measuring rod (Ano Sayol SL, Barcelona, Spain) and weight with a mechanical scale (Asimed T2, Barcelona, Spain). Body mass index (BMI) was calculated as weight (kg) /height (m2) following Shephard’s protocol (24).

#### Primary outcomes: characteristics of headache episodes

According to the IHS (25) the duration of the headaches was measured in hours per day, the intensity of pain with a visual analog scale (VAS) from 0 to 100, and the frequency of the episodes in days per month.

Another primary outcome present in the trial registration was the HIT-6 questionnaire, but this variable could not be collected.

#### Muscle thickness

Measurements of the thickness of the neck stabilizing muscles flexor longus colli and multifidus at the cervical C5-C6 level, respectively, were measured by ultrasonography (GE Healthcare, Chicago, Illinois, EE.UU.) at rest and counter-resistance. At rest, participants were asked to remain relaxed when taking the measurement, which was checked with palpation. In the counter resistance measurement, the participants were asked to perform a “double chin,” or cervical extension, depending on the muscle measured. For this protocol, the subjects previously performed a familiarization with the physiotherapist (26).

#### Craniocervical flexion test

The craniocervical flexion test (CCFT) was carried out to analyze the functionality of the cervical musculature, using the Stabilizer Pressure biofeedback, (Chattanooga Group, Hixon, EE.UU.), following the study of Thongprasert and Kanlayanaphotporn. The participants were placed supine with both knees bent and the cervical spine in a neutral position. The forehead and chin were horizontally aligned with the surface of the socket. The Stabilizer was be placed behind the neck in the suboccipital region before being inflated to the initial pressure of 20 mmHg. The participants were instructed to perform a slow and controlled craniocervical flexion in a nodding action, progressively increasing the pressure in 2 mmHg increments from 20 to 30 mmHg and maintaining it in each increment for 10s to 30s rest was allowed between successful raises. During testing, the contraction of the superficial neck flexor muscles was palpated by an investigator. The activation score was recorded, which is defined as the highest-pressure level change the participants could achieve and maintain steadily for 10 s (27).

#### Range of motion

The cervical ROM (CROM) was analyzed with a CROM goniometer Baseline (Fabrication Enterprises Inc., New York, EE. UU.). The movements evaluated were left rotation, right rotation, flexion, extension, left tilt, and right tilt, all measured in degrees. The participants remained in a sitting position to perform the measurements. The CROM goniometer is a reliable instrument that shows good inter-rater and intra-rater agreement (28). The test–retest reliability of the measurements made with the CROM was verified, with ICC values for all cervical measurements ranging between 0.89 (CI: 0.73–0.96) for flexion and 0.98 (CI: 0.95–0.99) for extension (29).

#### Pain pressure threshold

A FORCE DIAL FDK/FDN 100 algometer (Wagner Instruments, Greenwich, United States) was used to measure the pain pressure threshold (PPT) (kg/cm^2^), which is the most validated mechanical threshold at present (30). PPT measurements were performed bilaterally by a physiotherapist in the following regions: upper trapezius, masseter, temporalis, anterior tibialis, and median nerve in the flexion crease of the elbow. The participants, lying down and relaxed in the supine position, were informed that the investigators would perform mechanical pressure on different regions of the body to analyze the PPT and when they noticed pain, they should stop the investigators immediately to stop exerting said pressure, and to be able to quantify it through the measurement of the algometer, this procedure was carried out twice in order to perform the average between both measurements to obtain a more optimal result. The PPT measurements using algometry showed good-to-excellent interrater reliability (ICC: 0.64–0.92) and test–retest reliability (ICC: 0.72–0.95) (31).

All the variables were performed by a research team made up of six physiotherapists, two of them specialized in TTH. Each of the assessments was performed by at least two of them, to avoid any bias and poor execution of the techniques.

### Exercise intervention

Intervention group: The participants performed specific exercises to strengthen the craniocervical, shoulder girdle and shoulder muscles designed by a physiotherapist expert in therapeutic exercise and who oversaw teaching the participants how to perform the exercises correctly.

Apart from the pain, the main objective of this study was to increase the thickness of the neck muscles. The program duration was 12 weeks. All the exercises were performed 2 days a week during the first 6 weeks and 3 days a week for the remaining 6 weeks with at least 48 h of rest between sessions. The intensity of the exercises was 7 out of 10 on a Borg Scale of effort, which means “Hard. Vigorous. Strong effort needed.” The Borg Scale of effort allows to measure on a scale of 0 to 10 the participants’ perceived exertion (0 being the lowest, 10 the highest). The participants completed 3 sets of 8–10 reps each set.

**Figure 1:**
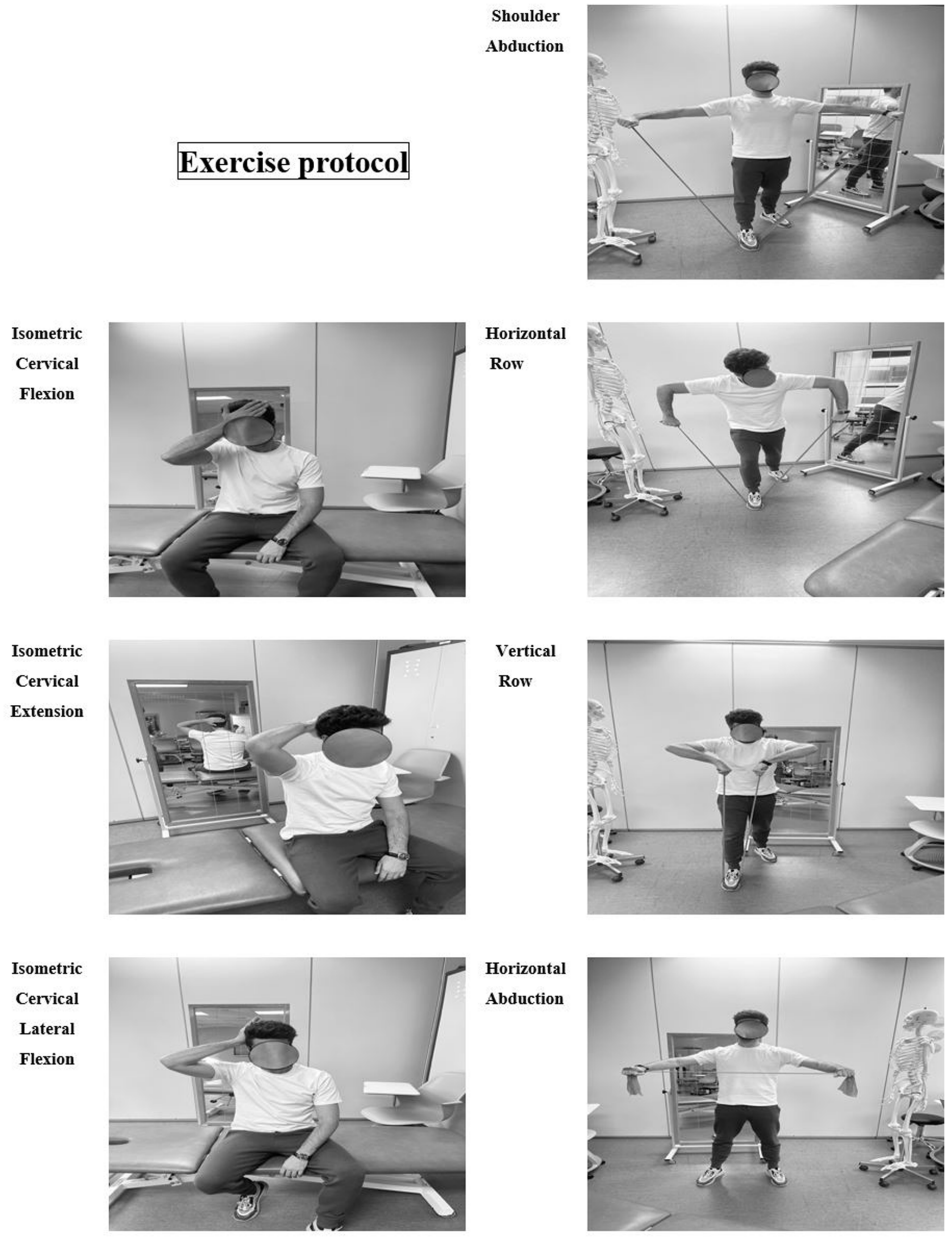
Exercise protocol.

First, a joint mobility warm-up consisting of mobility of the arms and cervical spine was performed. Then, the following shoulder exercises were performed with a TheraBand: shoulder flexion, shoulder abduction, horizontal row, bird exercise for shoulder, horizontal row and vertical row not overpassing 60 degrees internal rotation. The participants used TheraBand of different resistance levels, according to their muscle strength (see Figure 1).

Then, the participants performed the following isometric strengthening exercises of the cervical musculature: left and right flexion, extension, and lateral inclination. These movements were resisted by the patient’s hand for 6 sec.

The participants performed the exercise program without supervision, but once a week they hold a meeting with the physiotherapist to assess the execution of the exercises.

Throughout this process, the CG was told to continue with its daily activities without monitoring.

### Place of the intervention and moments of assessment

The participants performed the intervention at their homes. The initial assessments (PRE) were performed before starting the intervention, and the final assessments (POST) just after completing the exercise intervention.

### Statistical analysis

Descriptive demographic and clinical characteristics were collected, using means and standard deviation (SD). First, the Shapiro–Wilk test was performed to analyze the normality of the variables. Then, to analyze the homogeneity of the IG and CG in terms of age, BMI, and headache’s characteristics, an independent samples T-test (for parametric variables) and the Mann-U Whitney test (for non-parametric variables) were used. Finally, a two-way repeated measures analysis of covariance (ANCOVA) was performed to determine the effects of the intervention. In addition, the Eta partial squared was used as a measure of effect size, considering 0.01 a low effect size, 0.06 a moderate effect size, and 0.14 a large effect size. Finally, a Bonferroni correction was applied to correct multiple comparisons. The level of statistical significance was set at *p* < 0.05. All statistical analysis were performed with SPSS 25.0 (IBM, Armonk, NY, United States).

## Results

### Recruitment

**Figure 2:**
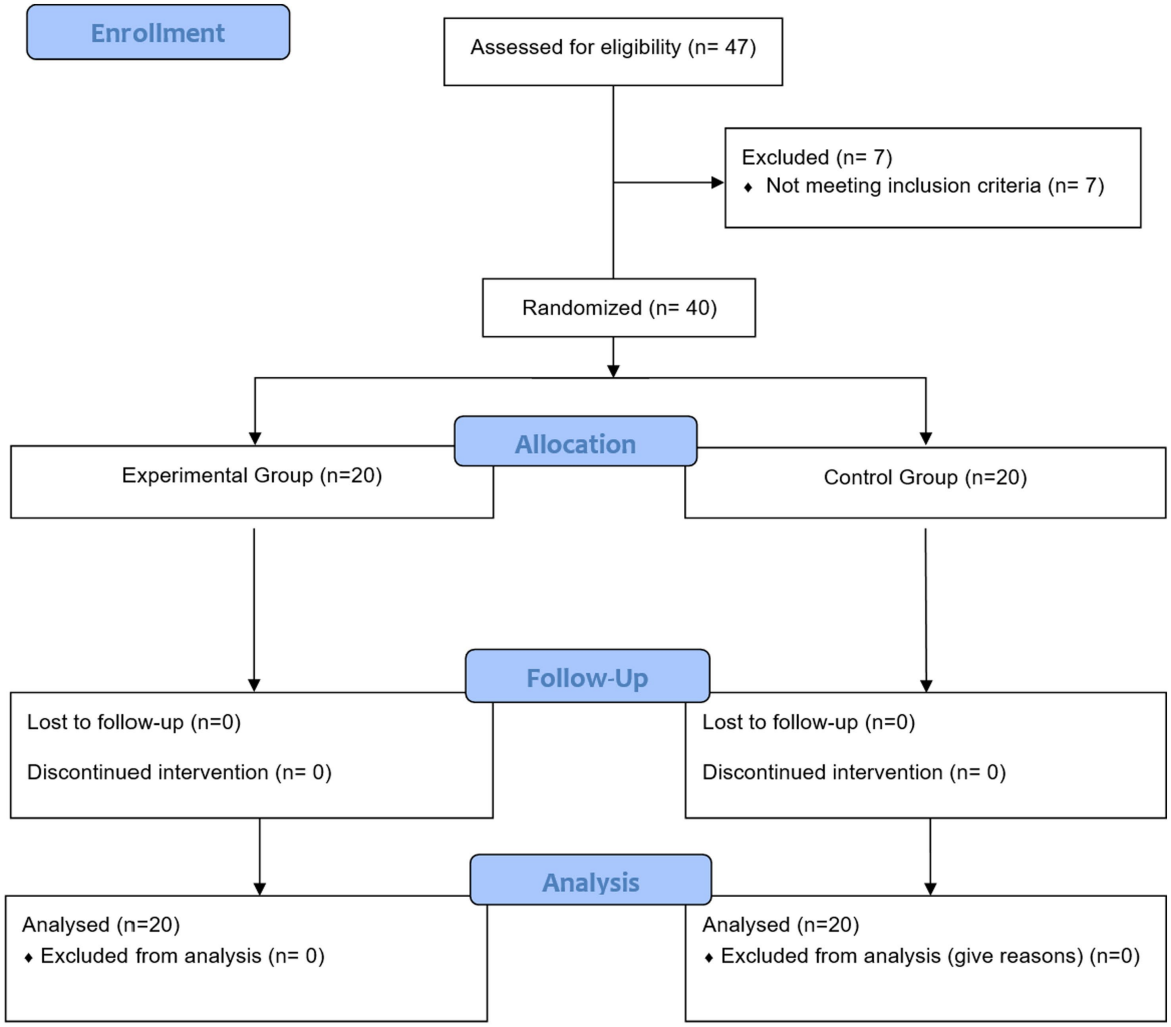
CONSORT flow diagram of an exercise intervention with tension-type headache patients.

Of 47 initially recruited patients, seven (15%) did not meet the inclusion criteria. The remaining 40 subjects were randomly assigned into two groups, IG (*n* = 20) and CG (*n* = 20). There were no dropouts, so the final analysis was performed on 20 CG and 20 IG participants, as shown in the flow diagram (Figure 2). There was no missing data. This study ended after the 12 weeks-exercise intervention to assess the effectiveness of the exercise program.

### Description of the sample

In the IG, 85% of the participants were female and 15% male, while in the CG, 75% were female and 25% were men. The BMI and the age of the participants are described in Table 1. There were no significant differences between groups in these variables at baseline.

**Table 1:** Baseline demographic characteristics.

	Intervention group (*n* = 20; 85% female)	Control group (*n* = 20; 75% female)	Value of *p*
Age (years)	33.9 ± 12.2	40.1 ± 14.0	0.147
Body Mass Index (kg/m^2^)	24.4 ± 3.9	24.2 ± 4.2	0.912

### Results of the specific cervical muscles exercise intervention

#### Headaches’s characteristics

As a result of the exercise intervention, compared to the CG, the IG participants significantly reduced the intensity (6.9 ± 1.3 PRE vs. 5.1 ± 2.1 POST; *p_txg_* = 0.001; *η^2^p* = 0.296), and the duration of the headaches (18.3 ± 7.5 PRE, vs. 11.3 ± 8.7 days per month POST; *p_txg_* = 0.007, *η^2^p* = 0.217) with a large effect size for these two variables. Regarding the frequency of the headaches, there were no significant differences between groups (*p_txg_* = 0.109; see Table 2).

**Table 2:** Effectiveness of the specific exercise intervention on headache characteristics, cervical muscles strength and thickness, cervical range of motion, and pressure pain threshold.

Variables	Group	PRE Mean ± SD [95% CI]	POST Mean ± SD [95% CI]	Value of *p* txg	*η^2^p* txg
Headache characteristics					
(Primary outcomes)					
Frequency of the headaches (days/month)	IG	10.1 ± 9.5 [5.8, 13.8]	7.4 ± 7.7 [3.4, 10.8]	0.109	0.083
	CG	10.5 ± 7.2 [6.1, 15.8]	10.1 ± 7.9 [6.0, 14.9]		
Duration of the headaches (hours)	IG	18.3 ± 7.5 [14.8, 22.3]	11.3 ± 8.7 [7.3, 15.5]	*0.007	0.217
	CG	14.0 ± 8.3 [9.2, 18.2]	13.6 ± 8.6 [8.5, 18.4]		
Intensity of the headaches (VAS)	IG	6.9 ± 1.3 [6.4, 7.7]	5.1 ± 2.1 [4.1, 5.9]	*0.001	0.296
	CG	7.1 ± 1.6 [6.1, 7.5]	6.9 ± 1.6 [5.8, 8.0]		
Secondary Outcomes					
Cervical muscles thickness (cm)					
Right multifidus (relaxed)	IG	1.1 ± 0.2	1.2 ± 0.2	*0.044	0.129
	CG	1.2 ± 0.2	1.2 ± 0.2		
Right multifidus (contracted)	IG	1.2 ± 0.3	1.3 ± 0.3	*0.001	0.297
	CG	1.4 ± 0.2	1.4 ± 0.2		
Left multifidus (relaxed)	IG	1.1 ± 0.2	1.2 ± 0.2	0.166	0.063
	CG	1.2 ± 0.3	1.2 ± 0.2		
Left multifidus (contracted)	IG	1.3 ± 0.3	1.3 ± 0.3	0.127	0.076
	CG	1.4 ± 0.3	1.4 ± 0.3		
Right deep flexors (relaxed)	IG	0.9 ± 0.2	1.0 ± 0.2	0.092	0.092
	CG	1.0 ± 0.2	1.0 ± 0.2		
Right deep flexors (contracted)	IG	1.2 ± 0.2	1.3 ± 0.2	* < 0.001	0.474
	CG	1.2 ± 0.2	1.1 ± 0.2		
Left deep flexors (relaxed)	IG	0.9 ± 0.2	1.0 ± 0.2	0.118	0.08
	CG	1.0 ± 0.2	1.0 ± 0.2		
Left deep flexors (contracted)	IG	1.2 ± 0.2	1.3 ± 0.2	*0.003	0.251
	CG	1.2 ± 0.2	1.2 ± 0.2		
Cervical range of motion (degrees)					
Flexion	IG	60.3 ± 13.4	64.3 ± 12.9	0.361	0.028
	CG	62.5 ± 10.5	63.5 ± 10.4		
Extension	IG	63.1 ± 9.0	65.1 ± 6.2	0.78	0.003
	CG	56.2 ± 10.7	57.4 ± 9.7		
Rotation	IG	128.3 ± 16.2	132.4 ± 12.1	0.31	0.033
	CG	126.5 ± 12.4	126.1 ± 14.1		
Lateral flexion	IG	73.3 ± 12.7	81.4 ± 18.3	*0.002	0.269
	CG	70.9 ± 8.0	71.7 ± 10.1		
Pain pressure threshold (kg/cm^2^)					
Right temporalis	IG	2.5 ± 0.7	2.9 ± 0.7	* < 0.001	0.392
	CG	2.8 ± 0.9	2.6 ± 0.8		
Left temporalis	IG	2.3 ± 0.7	3.0 ± 0.7	* < 0.001	0.341
	CG	2.5 ± 0.9	2.5 ± 0.9		
Right upper trapezius	IG	3.0 ± 0.8	3.6 ± 0.8	*0.047	0.125
	CG	3.8 ± 1.5	3.6 ± 1.5		
Left upper trapezius	IG	3.3 ± 1.0	3.8 ± 1.0	*0.022	0.163
	CG	3.6 ± 1.7	3.3 ± 1.3		
Right masseter	IG	1.9 ± 0.6	2.4 ± 0.6	0.165	0.063
	CG	2.1 ± 0.8	2.2 ± 0.7		
Left masseter	IG	1.9 ± 0.5	2.5 ± 0.6	* < 0.001	0.332
	CG	2.3 ± 1.0	2.1 ± 0.6		
Right median nerve	IG	3.4 ± 1.1	3.8 ± 1.1	0.184	0.058
	CG	3.7 ± 1.3	3.3 ± 1.1		
Left median nerve	IG	3.5 ± 1.1	3.9 ± 1.0	*0.039	0.134
	CG	3.8 ± 1.4	3.3 ± 0.9		
Right tibialis	IG	6.3 ± 1.0	7.0 ± 0.5	0.891	0.001
	CG	6.3 ± 2.1	6.8 ± 1.7		
Left tibialis	IG	6.7 ± 0.3	7.2 ± 0.7	0.861	0.001
	CG	6.5 ± 1.9	6.9 ± 1.7		
Cervical flexion test (mm Hg)	IG	20.8 ± 1.2	23.7 ± 1.5	* < 0.001	0.464
	CG	21.3 ± 1.8	21.5 ± 1.8		

IG, intervention group; CG, control group; *η*^2^ *p* txg: effect size time x group; CI: confidence interval; SD: Standard Deviation. Differences between groups were evaluated using two-way repeated measure ANCOVA with Bonferroni. Significance was set at < 0.05. Bold values means: statistically significant difference between groups.

#### Strength of the deep cervical flexors

After the exercise intervention, compared to the CG, the IG participants significantly improved the strength in the cervical flexion test (*p_txg_* < 0.001), with a large effect size.

#### Thickness of the deep cervical muscles

After the specific strength exercises intervention, compared to the CG, the IG participants significantly improved the thickness of the right multifidus (relaxed *p_txg_* = 0.044; and contracted (*p_txg_* < 0.001), right deep flexors contracted (*p_txg_* < 0.001), and left deep flexors contracted *p_txg_* = 0.003), with a large effect size at all times.

#### Cervical range of motion

There were no differences between groups in the cervical ROM, except in the case of lateral tilt, where the IG showed a significant improvement (*p_txg_* = 0.002), with a large effect size.

#### Pressure pain threshold

Compared to the CG, the IG participants significantly improved the PPT of the right and left temporalis (*p_txg_* < 0.001 in both cases), right and left upper trapezius (*p_txg_* = 0.047 and 0.022, respectively), left masseter (*p_txg_* < 0.001), and left median nerve (*p_txg_* = 0.039), with a large effect size in all cases.

#### Compliance with the exercise intervention

The compliance with the intervention was high among the IG, with a mean attendance at the training sessions of the physical exercise intervention of 87%. There were no adverse effects caused by the intervention.

## Discussion

The objective of the present study was to examine the effectiveness of a targeted non-supervised 12-week strength training in TTH patients, based on the theoretical influence of muscular impairments at the craniocervical and shoulder region, cervical ROM, cervical flexors endurance, and PPT of different muscles.

The main findings of the present study were that the participants of the intervention group experienced significant changes of medium-large magnitude after 12 weeks in: (i) headache characteristics resulting in a decrease of the pain intensity and duration; (ii) cervical muscle thickness by the gain of multifidus and longus colli thickness at rest and contraction; (iii) cervical ROM resulting in an increase of cervical lateral inclination; (iv) PPT resulting in an increase of temporalis, masseter, upper trapezius muscle and median nerve; and (v) CCFT by the gain of deep cervical flexor muscles endurance, compared with the CG during the same period of time.

Regarding current scientific evidence of strength training in patients with TTH, a recent systematic review of randomized controlled trials demonstrated that strength exercise may decrease pain intensity, duration, and frequency, with a moderate clinical effect (18). In the present study, the IG significantly reduced the pain intensity and duration compared to the CG, with a large effect size, although no differences were observed in the frequency of the headache’s episodes. Therefore, our results partially support that strength training has beneficial effects on pain characteristics.

Another finding of our study was that the IG showed a significant increase of the right cervical multifidus and both sides of longus coli muscle thickness during contraction but not at rest. To the best of authors’ knowledge, this is the first study that has examined the effectiveness of strength training on cervical muscles thickness. Muscle strength has been associated with muscle thickness (32). Thus, Madsen et al. (33) showed that patients with TTH exhibited decreased muscle strength in the neck extensor muscle and cervical extension/flexion ratio compared to healthy people. Therefore, according to our findings, strength training can improve muscle thickness in patients with TTH and may result in a decrease of disability (18). Further research in needed to confirm our findings.

In the present study, only the lateral inclination ROM increased in the IG, while the cervical flexion, extension and rotation ROM did not show any changes compared to the CG. At baseline, both groups have reduced cervical ROM, according to normative values (28). In this line, Liang et al. (34) showed that people with TTH had less cervical ROM compared to healthy controls. In addition, several studies have investigated the effectiveness of different manual therapies such as cervical mobilization or myofascial techniques on cervical ROM (35–37), while no studies have investigated yet the effects of shoulder/neck strengthening on cervical ROM in patients with TTH. Therefore, in future studies the objective of the treatment should focus on a multimodal approach (e.g., manual therapy and strength exercise) to investigate the effects in cervical ROM.

In relation to the measures at different points (craniocervical, median nerve and tibialis muscle), we hypothesized that exercise intervention may increase PPT of these structures. Although no differences were found between groups for the median nerve (only left median nerve PPT) and tibialis muscle, a significant improvement in mechanical sensitivity for the upper trapezius, masseter and temporalis muscle was observed. In the present study, the baseline PPT values on the different craniocervical muscles (upper trapezius, masseter and temporalis) of the patients were slightly higher than those previously reported (38–40). PPT measures have been used in following up the efficacy of different treatments. The improvement obtained in our study for the craniocervical muscles PPT was similar to those obtained previously in patients with different pain conditions. For example, strength exercise of the neck and shoulder region diminished pain and was associated with decreased mechanical pressure in women with neck pain (39). As theorized previously, strength training may increase the Na + and K+ pumps and adenosine triphosphate concentration in the neck muscles involved (41). Although we hypothesized that our patients would benefit from strength exercise, we cannot conclude that a strength exercise program can decrease mechanical sensitivity to pressure in peripheral sensitization points (median nerve and tibialis muscle). The role of peripheral and central mechanisms has been previously discussed (42) and these mechanisms should not be ignored. Therefore, further research should assess the effects of different treatment modalities in patients with TTH and signs of peripheral or central sensitization.

As expected, a significant increase in craniocervical muscles endurance was found in the IG compared to the CG. At baseline, both groups showed lower values for CCFT than previous studies (43, 44), in accordance with the results observed in other studies (45). Moreover, muscle pain has been associated with lower muscle strength (33). In contrast with previous research (46), after performing craniocervical exercises the IG showed a significant increase in deep cervical flexor muscles compared with controls. This data suggests that isometric training of cervical region improves test performance.

The strength of the present study is that the IG performed a simple and cheap physical training with elastic resistance bands, with clinically relevant results. Varangot-Rielle et al. (18) demonstrated that a regular strength or aerobic intervention that incorporates individualized and graded activity exposure may improve pain and disability in people with primary headache such as TTH or migraine.

However, the present study has several limitations: (i) the lack of a detailed training diary did not allow us to record headache pain before, during and after training; (ii) a color registration and length of elastic resistance bands during the intervention period could have demonstrated more precisely the resistance progress; (iii) the lack of blinding of participants of both groups; and (iv) we did not assess other baseline factors such as psychological (e.g., kinesiophobia or pain catastrophizing) or headache-related impact that may have interfered in our results.

## Conclusion

A 12-week strength training of neck and shoulder region induced positive changes in pain intensity and duration, neck muscle thickness, lateral inclination ROM, craniocervical PPT and CCFT in patients with TTH. Future interventions are needed to investigate if normalization of pain characteristics and markers of craniocervical strength can more meaningfully impact headache control.

## Clinical implications

- Patients with chronic tension-type headache should perform specific strength training of cervical and shoulder muscles to decrease pain intensity and duration of the headaches.
- The thickness of deep cervical muscles, the strength of deep cervical flexors, and the pain pressure threshold of specific muscles should be assessed in patients with tension-type headache.

## Data availability statement

The raw data supporting the conclusions of this article will be made available by the authors, without undue reservation.

## Ethics statement

The studies involving humans were approved by the current study was approved by the Research Ethics Committee of the Rey Juan Carlos University of Madrid (reference number: 1802202105721). The studies were conducted in accordance with the local legislation and institutional requirements. The participants provided their written informed consent to participate in this study. Written informed consent was obtained from the individual(s) for the publication of any potentially identifiable images or data included in this article.

## Author contributions

DM-V: Conceptualization, Investigation, Visualization, Formal analysis, Validation, Writing – original draft. AS-S: Conceptualization, Writing – review & editing. ÁG-d-lF: Formal analysis, Investigation, Writing – review & editing. GG-P-d-S: Data curation, Methodology, Supervision, Writing – original draft. DD-B: Supervision, Validation, Writing – original draft. Jd-B-M: Validation, Writing – original draft, Writing – review & editing.
